# Agreement between Ultra-Short-Term and Standard Heart Rate Variability Analysis in Resting and Post-Exercise Conditions

**DOI:** 10.3390/life14070837

**Published:** 2024-06-30

**Authors:** Kai Jin, Zhenxiang Guo, Zining Qiao, Meng Liu, Yi Yang, Changnan Xu

**Affiliations:** 1 Department of Physical Education, Southeast University, Nanjing 210096, China; 101009989@seu.edu.cn; 2 Sports Coaching College, Beijing Sport University, Beijing 100084, China; guozhenxiang@bsu.edu.cn (Z.G.); liusky@bsu.edu.cn (M.L.); 3 School of Strength and Conditioning Training, Beijing Sport University, Beijing 100084, China; qiaozining@bsu.edu.cn; 4 College of Physical Education, Hengxing University, Qingdao 266100, China; 5 Physical Education Department, Nanjing University of Aeronautics and Astronautics, Nanjing 210016, China

**Keywords:** heart rate variability, ultra-short-term, standard short-term, rest, exercise

## Abstract

Background: Short-term (5 min) heart rate variability (HRV) analysis is widely used in assessing autonomic nervous system activity during exercise. While shortening the HRV measurement duration can help improve its application efficiency, its accuracy needs to be verified. This study investigated the agreement between ultra-short-term (UST) HRV (3 or 4 min) and standard 5 min HRV and explored the optimal recording duration under resting and post-exercise conditions. Methods: Fourteen participants exercised on a cycle ergometer at 60% of their maximum peak power. Data were collected during the rest condition (Pre-E) and three post-exercise conditions (Post-E1, Post-E2, and Post-E3), with indicators of the standard deviation (SDNN) of the ultra-short and short-term RR intervals and the root mean square (RMSSD) of the continuous difference between RR intervals. Repeated measures ANOVA, Cohen’s d statistic, Bland–Altman analysis, and interclass correlation coefficients (ICC) assessed the agreement between UST-HRV and ST-HRV. Results: The consistency results of SDNN and RMSSD in resting and post-exercise were different. At the Pre-E, Post-E2, and Post-E3 phases, no statistical differences for SDNN and RMSSD were observed, with ICCs surpassing 0.9, indicating a high level of agreement. However, at Post-E2, there was a significant difference between 3 min RMSSD and 5 min RMSSD (*p* < 0.05), as well as between 3 min SDNN, 4 min SDNN, and 5 min SDNN (*p* < 0.05). Furthermore, the limits of agreement were observed to decrease as the time duration increased in Bland–Altman plots. Conclusions: UST-HRV analysis is a reliable substitute for standard 5 min HRV assessment, particularly during resting conditions. For post-exercise measurements, assessing the appropriateness of a 3- or 4 min duration based on the exercise’s length is recommended to ensure accuracy and reliability.

## 1. Introduction

Heart rate variability (HRV) refers to the variations in each cardiac cycle, which can reflect the regulatory information of neurohumoral factors on the cardiovascular system [1]. HRV parameters can be altered by both infectious diseases, such as COVID-19, and lifestyle factors [2,3]. Consequently, the assessment of HRV is crucial for prognostication, as these parameters hold significant relevance in contemporary clinical practice. It also serves as a key physiological indicator for understanding exercise performance [4]. With the popularization of exercise physiological monitoring equipment, HRV has become an important tool for evaluating autonomic nervous system function during exercise. Research indicates that standard short-term HRV (ST-HRV), typically spanning a 5-min recording period, reliably reflects autonomic regulation [5]. Moreover, shorter data cycles in HRV measurement offer benefits such as improved time efficiency in data collection and enhanced practicality for physiological monitoring applications [6]. This prompts the question: Can we further shorten the recording period for HRV measurement? However, the effectiveness of ultra-short-term HRV (UST-HRV), typically with a recording period of less than 5 min, remains a topic of debate [7]. There is a certain degree of consistency in the evaluation results of UST-HRV and ST-HRV under different physiological states, yet comprehensive analysis in post-exercise scenarios is limited [8].

Specifically, some studies have demonstrated a strong correlation and consistency between UST-HRV and ST-HRV in resting conditions, advocating for shortening HRV measurement time to improve efficiency [9,10,11]. However, continuously shortening the time may result in the loss of some information and reduce the reliability of the measurement [12]. Therefore, it is necessary to explore the optimal duration for measuring UST-HRV under resting conditions. Additionally, studies have suggested that HRV parameters decrease as exercise duration and intensity increase during exercise [13], and the parasympathetic activity related with HRV becomes more suppressed during the post-exercise recovery period [3]. This emphasizes the importance of HRV analysis in the post-exercise period. However, to our knowledge, these studies have primarily relied on ST-HRV, overlooking research on UST-HRV. Therefore, it is necessary to investigate cardiovascular autonomic modulation using UST-HRV to verify its effectiveness [14,15]. 

To sum up, existing studies have elucidated the consistency between UST-HRV and ST-HRV in resting conditions, but there is a notable research gap regarding the post-exercise recovery phase. This gap is particularly relevant due to the dynamic changes in autonomic activity following physical exertion. Addressing this, our study investigated the consistency between UST-HRV measures (0–3 min and 0–4 min) and the ST-HRV at multiple post-exercise recovery phases [16]. Furthermore, our study also explored the optimal recording duration for HRV analysis under the rest and post-exercise conditions, an aspect not comprehensively studied in previous works [17]. Therefore, the first aim of this study is to examine the relationship between UST-HRV and ST-HRV under rest and post-exercise conditions, and the second aim is to explore the optimal period recording for obtaining reliable HRV parameters, establishing evidence that UST-HRV analysis is a valuable method for autonomic evaluation under rest and post-exercise conditions.

## 2. Materials and Methods

### 2.1. Participants

The sample size was calculated using Gpower (version 3.1.9.7, Franz Faul, University of Kiel, Kiel, Germany), with an effect size of f = 0.40, an α err prob of 0.05, and a power (1-β err prob) of 0.80 for ANOVA (repeated measures, within factors) [18], calculating that the total necessary sample size was 10. For this study, 20 healthy trained males (Tier 2 [19]) of Han Chinese ethnicity were recruited through voluntary participation in this study. Anthropometric measurements, including age, height, weight, body mass index (BMI), and resting heart rate (HR), were collected for all participants (Table 1). Eligible participants were required to have no family history of cardiovascular disease and not be taking any medication. Additionally, participants were requested to abstain from alcohol, coffee, or any other performance-affecting beverages for a period of 24 h prior to this study. Furthermore, all participants provided written informed consent. Participants were informed that their participation in this study would not affect their employment status. The study protocol was in accordance with the Declaration of Helsinki and was approved by the Ethics Committee of Beijing Sport University (2023073H).

### 2.2. Experimental Protocols

The experimental sessions were conducted in a distraction-free room with a controlled temperature of 22–24 °C. Participants rested quietly in a seated position for 10 min before data collection. Baseline heart signals were then recorded for 8 min (Pre-E). A 3-min warm-up on a cycle ergometer followed, to familiarize participants with the protocols, ensure sensors were properly attached, and verify data collection.

**Figure 1:**
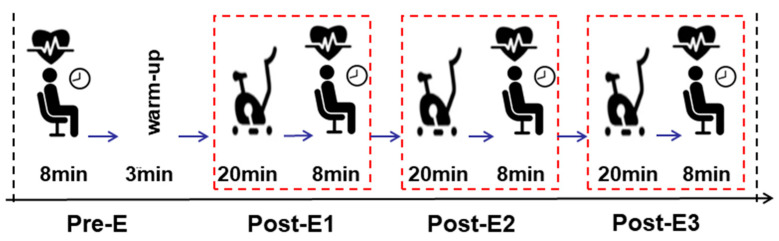
The procedure of the experimental protocol.

Participants then performed the cycle ergometer exercise at the same intensity level for three trials, each lasting 20 min with an 8-min break in between. HRV signals were collected immediately after each exercise trial, lasting about 8 min. Four time points were used to collect HRV signals: Pre-E, Post-E1, Post-E2, and Post-E3. Participants were instructed to remain stable, breathe naturally, and sit still to avoid motion interference with the HRV signals. The experimental protocol is illustrated in Figure 1.

### 2.3. Data Collection

HRV metrics were obtained as previously described [18]. A portable telemetric HR monitoring system was used to record HRV (Polar team Pro, Polar Electro, Kemple, Finland). Each player was provided with an HR sensor and an HR belt for the entire duration of the experiment. For HRV analysis, only normal-to-normal (NN) intervals were included. Ectopic beats and artifacts were identified and excluded using Kubios HRV Premium (ver. 3.2) (The Biomedical Signals Analysis and Medical Imaging Group, University of Kuopio, Finland), ensuring that the HRV metrics were derived from sinus rhythm intervals only. The artifact correction threshold was established at a moderate level, with a window width of 300 s and a window overlap of 50%. Detrending methods utilized smoothing priors set at a Lambda value of 500 [12]. If the percentage of ectopic beats in the daily measurements exceeded 5%, the data were excluded from the analysis. Time-domain HRV parameters, such as the standard deviation (SDNN) and root mean square (RMSSD), were measured and analyzed during specific time periods within each 5-min heart rate recording interval, including (1) 0–3 min and 0–4 min (UST-HRV) and (2) 0–5 min (ST-HRV). 

### 2.4. Statistical Analysis

Statistical analysis was conducted using SPSS 26.0 (version 26.0; SPSS Inc., Chicago, IL, USA). Quantitative parameters were presented as mean ± standard deviation (Mean ± SD). The Shapiro–Wilk Normality Test was applied to evaluate the distribution of HRV parameters. Repeated one-way ANOVA with post hoc Bonferroni test compared UST-HRV to ST-HRV. Cohen’s d values were calculated and used to determine the effect size (ES), with interpretations categorized as trivial (<0.2), small (0.2–0.6), moderate (0.6–1.2), large (1.2–2.0), or very large (>2.0) [20]. The intraclass correlation coefficient (ICC) assessed the agreement between UST-HRV and ST-HRV, with ICC values < 0.4 considered small, 0.4–0.75 moderate, 0.75–0.9 large, and >0.9 perfect [20]. Bland–Altman plots identified upper and lower limits of agreement for SDNN and RMSSD between ultra-short and standard time analysis [21]. Results with *p* values < 0.05 were considered statistically significant.

## 3. Results

As summarized in Table 2, the Mean ± SD values of SDNN and RMSSD from UST and ST-HRV analysis were obtained at rest and for three post-exercise trials. We compared the difference of SDNN and RMSSD between ultra-short time analysis and standard time analysis, respectively. At the Pre-E, Post-E2, and Post-E3 phases, no statistical differences for SDNN and RMSSD were found. However, at the Post-E1 phase, statistical differences were noted. The 3 min RMSSD suggested a significant difference compared to 5 min RMSSD. Similarly, 3 min SDNN and 4 min SDNN demonstrated significant differences compared to 5 min SDNN.

Table 3 provided the ICC values between UST-HRV and ST-HRV for four trials. At Pre-E, the ICCs for UST-HRV parameters were deemed nearly perfect, surpassing 0.9. At Post-E1, ICCs between 3 min SDNN/4 min SDNN and 5 min SDNN indicated moderate agreement (ICC = 0.86/ICC = 0.89), and those of 3 min SDNN/4 min SDNN showed perfect consistency in comparisons (ICC = 0.98/ICC = 0.99). Notably, as the exercise duration increased, there was no significant decrease in ICC values observed throughout the trail. In the three post-exercise trials, SDNN ICC increased with the HRV measurement duration, with 4 min HRV demonstrating high values and nearly perfect reproducibility in all comparisons (ICCs > 0.95). 

In Table 4, Cohen’s d values were calculated to quantify the bias in SDNN and RMSSD analyzed across different recording periods. Compared to 5 min SDNN, 3 min SDNN and 4 min SDNN showed small ESs at Post-E1 and Post-E3 (Cohen’s d = 0.28~0.41). In other comparisons, the ESs were trivial (Cohen’s d < 0.2).

**Figure 2:**
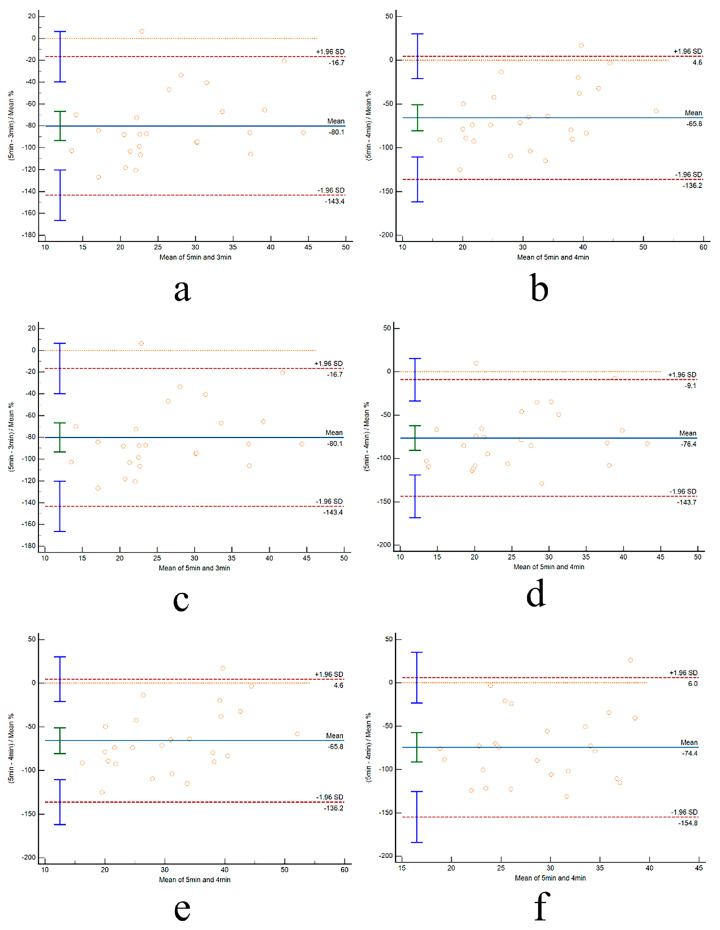
**Consistency for RMSSD between ultra-short duration and 5 min duration for different conditions.** (**a**) Pre-E 3 min–5 min; (**b**) Pre-E 4 min–5 min; (**c**) Post-1 3 min–5 min; (**d**) Post-1 4 min–5 min; (**e**) Post-2 3 min–5 min; and (**f**) Post-2 4 min–5 min. Each circle represents one participantn’s data.

**Figure 3:**
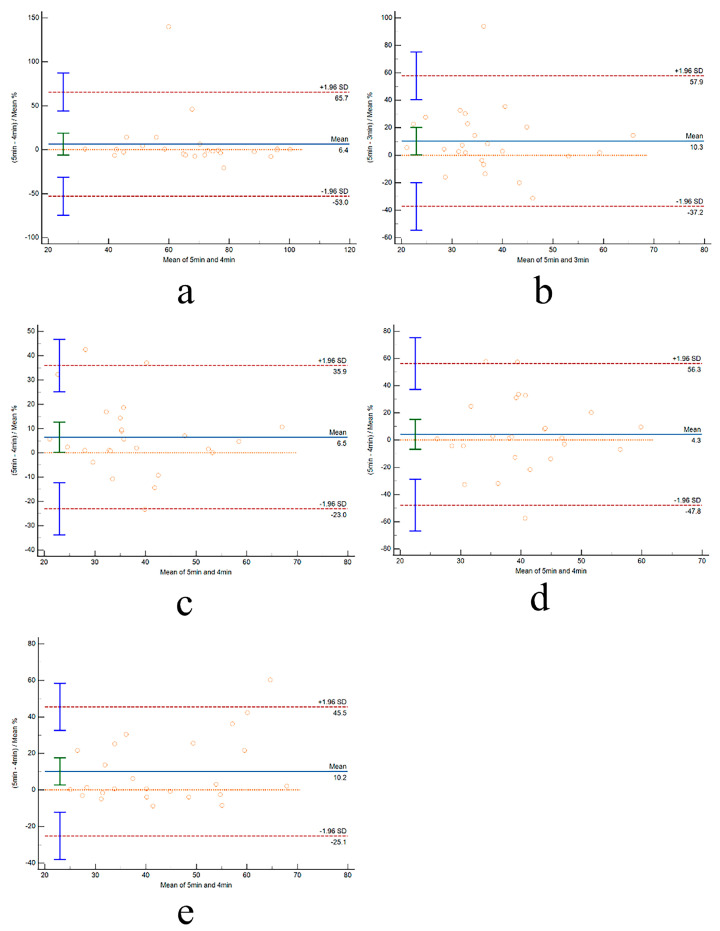
**Consistency for SDNN between ultra-short duration and 5 min duration for different conditions.** (**a**) Pre-E 4 min–5 min; (**b**) Post-1 3 min–5 min; (**c**) Post-1 4 min–5 min; (**d**) Post-2 3 min–5 min; and (**e**) Post-2 4 min–5 min. Each circle represents one participantn’s data.

As illustrated in Figure 2 and Figure 3, the consistency between UST-HRV and ST-HRV at rest and post-exercise was evaluated using Bland–Altman plots. These plots depicted the bias and the limits of agreement (±1.96SD) within their corresponding panels. Notably, the limits of agreement were observed to decrease as the time duration increased.

## 4. Discussion

This study compared UST-HRV with ST-HRV under both rest and three post-exercise conditions, revealing the consistency between 3 min HRV, 4 min HRV and 5 min HRV. Specifically, no significant differences were found between 3 min HRV, 4 min HRV and 5 min HRV at rest, with higher ICCs, suggesting strong agreement levels. These findings are consistent with previous studies [22,23], which indicated that HRV obtained from shorter periods (<4 min) had good agreement with that obtained from 5 min periods [24]. Additionally, in the second and third post-exercise conditions, no significant differences were observed between 3 min HRV, 4 min HRV, and 5 min HRV, with ICCs exceeding 0.9, indicating strong consistency. These consistency values confirmed the applicability of UST-HRV after aerobic exercise, which is consistent with a previous study [25]. However, there were significant differences between UST-HRV and ST-HRV under the first post-exercise condition, indicating the inconsistency when the recording period is shorter than 3 min. This phenomenon can be reasonably explained within the perspective of exercise physiology. Vagal withdrawal persisted after exercise, revealing the stability of vagal influence on heart rate during the initial early recovery period [22,23]. This stability may be attributed to the increased error of unstable RR-interval data post exercise. Thus, a 3-min duration for daily HRV monitoring under rest conditions could provide acceptable reliability, while a longer duration (3 min or more) was recommended to ensure the accuracy of HRV measurements after exercise. 

Furthermore, considering that RMSSD reflects vagal modulation and SDNN is influenced by both sympathetic and parasympathetic nerve activities, this study observed an increase in parasympathetic nerve activity and a decrease in sympathetic nerve activity during the recovery period after exercise. These findings are not only consistent with previous studies but also provide reliable information for using UST-HRV as a monitoring tool for autonomic alteration after exercise [26]. It was worth noting that frequency-domain parameters of HRV were not analyzed in this study due to the inadequate duration of ultra-short periods (<5 min) to conduct power spectrum analysis [27]. In this study, we also used Bland–Altman plots to assess the consistency of UST-HRV parameters and ST-HRV parameters in different time periods after exercise, aiming to validate the applicability of UST-HRV analysis. Our findings revealed a positive correlation between extended time periods and increased consistency. Specifically, we observed that SDNN and RMSSD exhibited high dependence on recording length, possibly because SDNN reflects the total power of HRV frequency components and RMSSD represents the high-frequency HRV components, both of which are influenced by recording duration [26]. In summary, our study highlighted the utility of UST-HRV as a standard HRV measurement alternative, and also determines the optimal ultra-short periods for HRV evaluation at rest and after exercise, respectively.

For cardiovascular health assessment, the autonomous regulation of heart rate after exercise is important [28], as extreme autonomous response of the cardiovascular system to vigorous exercise may increase the risk of cardiovascular disease and mortality [26,29]. Currently, the rising popularity of wearable devices based on HRV parameter monitoring underscores the significance of shortening the duration of HRV monitoring. This advancement could facilitate the development of wearable devices, enhance the convenience of HRV measurements, and increase its practicality in physical exercise. Additionally, timely monitoring of HRV during exercise could also provide guidance for adjusting exercise programs. 

This study has several limitations that should be noted. HRV metrics such as SDNN and RMSSD can vary significantly with different heart rates. Although this study primarily focused on the measurement duration of HRV, we acknowledge that heart rate is an important variable whose fluctuations can affect the stability of HRV metrics. Therefore, future studies should integrate heart rate measurements to more accurately assess HRV variations. The participant’s fitness and circadian rhythm can significantly influence SDNN values. In future studies, it would be beneficial to include assessments of participants’ fitness levels (e.g., VO2max test) and account for the time of day when measurements are taken to control for circadian variations. The results of HRV may be affected by age, race, and gender; future studies should include a diverse sample in terms of gender, age, and race to explore the interactions between these variables. Irregular rhythm may influence the results of HRV; future studies should incorporate methods to ensure the accuracy of HRV measurements by distinguishing between normal sinus rhythm and irregular rhythms. This could include the following: Utilizing electrocardiogram (ECG) monitoring to classify cardiac rhythms during data collection. Implementing rigorous screening protocols to exclude participants with known arrhythmias or irregular rhythms. Analyzing the data separately for participants with normal sinus rhythm and those with irregular rhythms to assess the impact on HRV measurements. With the advancement of Artificial Intelligence (AI), its role in the future perspectives on heart rate variability (HRV) parameters is becoming increasingly significant [30]. Consequently, future research should focus on enhancing the application of AI in the monitoring and computation of HRV.

## 5. Conclusions

The ultra-short time recordings for HRV analysis proved to be a reliable substitute for the standard 5 min assessment in evaluating autonomic activities. For daily HRV measurements under rest conditions, recording periods of 3 or 4 min were deemed acceptable. However, for HRV analysis at post-exercise, the duration of exercise needs to be taken into account. If the exercise duration exceeds 40 min, a recording time of 3 or 4 min is not acceptable, and vice versa, and a 5-min standard measurement is required. We strongly endorse the efficacy of UST-HRV in evaluating autonomic response both at rest and post-exercise. This study is expected to contribute to the development of HRV monitoring applications in daily life.

## Figures and Tables

**Table 1:** Anthropometric parameters of participants.

Age (Years)	Height (cm)	Weight (kg)	BMI (kg/m^2^)	HR (beats/s)
22.63 ± 1.53	179.42 ± 5.23	73.41 ± 5.64	22.80 ± 0.59	61.67 ± 4.20

**Table 2:** HRV parameters at ultra-short duration (3 min and 4 min) and 5 min timepoint.

	3 min	4 min	5 min
RMSSD			
Pre-E	41.3 ± 16.8	41.0 ± 16.1	40.5 ± 15.2
Post-E1	17.5 ± 9.8 *	18.1 ± 10.1	18.7 ± 10.5
Post-E2	20.6 ± 10.3	21.1 ± 11.1	21.6 ± 11.6
Post-E3	19.6 ± 9.5	20.3 ± 10.1	20.5 ± 10.5
SDNN			
Pre-E	63.7 ± 20.0	63.8 ± 19.6	65.9 ± 18.8
Post-E1	39.8 ± 16.6 *	43.0 ± 16.4 *	46.7 ± 17.1
Post-E2	42.8 ± 15.3	44.3 ± 18.6	44.3 ± 18.6
Post-E3	43.8 ± 16.0	46.0 ± 18.2	49.6 ± 19.6

* *p* < 0.05, compared with 5 min standard duration.

**Table 3:** ICCs of HRV parameters between ultra-short duration (3 min and 4 min) and 5 min standard duration for 4 conditions.

	3 min	4 min
RMSSD		
Pre-E	0.98	0.99
Post-E1	0.98	0.99
Post-E2	0.98	0.99
Post-E3	0.98	0.99
SDNN		
Pre-E	0.86	0.89
Post-E1	0.96	0.97
Post-E2	0.95	0.99
Post-E3	0.90	0.92

**Table 4:** Cohen’s d values for SDNN and RMSSD of different duration for 4 conditions.

	3 min	4 min
RMSSD		
Pre-E	−0.05	−0.03
Post-E1	−0.12	−0.06
Post-E2	−0.09	−0.04
Post-E3	−0.09	−0.02
SDNN		
Pre-E	−0.12	−0.11
Post-E1	−0.41 *	−0.28 *
Post-E2	−0.09	−0.01
Post-E3	−0.29 *	−0.16

* *p* < 0.05, compare with 5 min standard duration.

## Data Availability

The original contributions presented in this study are included in the article, further inquiries can be directed to the corresponding author.
